# Supplementary material for: Mitotic reactivation and transcriptional bursting govern transcriptional noise in the early Drosophila embryo
Source: Development. 2026 Apr 14;153(7):dev204953. doi: 10.1242/dev.204953 (PMC13120686; doi:10.1242/dev.204953)
Supplement: Supplementary information [file develop-153-204953-s1.pdf]

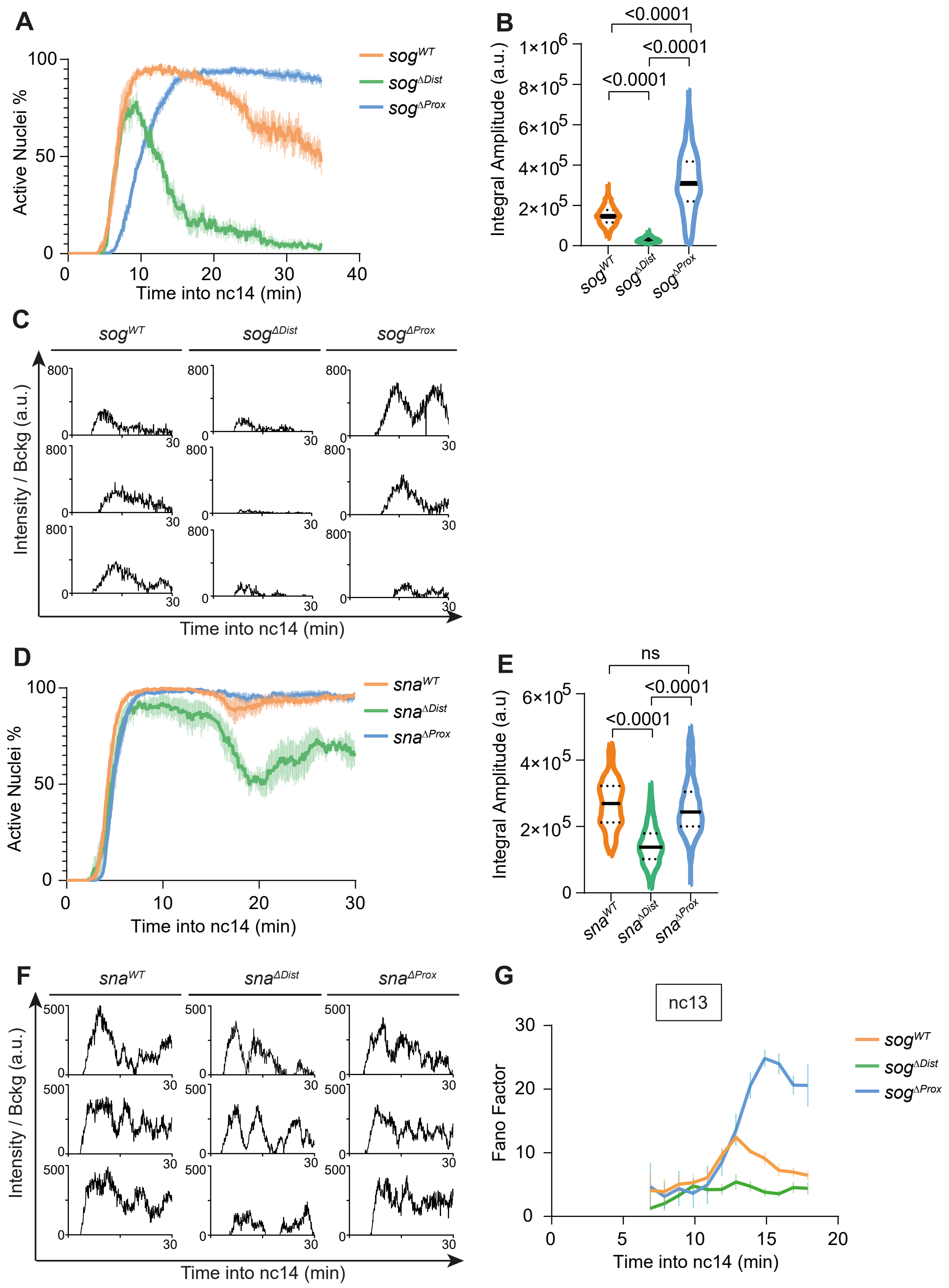

Fig. S1. Quantifying transcription dynamics of *sog-MS2* and *sna-MS2 BAC alleles* (A,D) Instantaneous active nuclei percentage (mean+s.e.m.) during nc14 **(B,E)** Distribution of AUC from intensity traces for the first 30 minutes of nc14. Solid line represents median, dashed lines are 1st and 3rd quartiles. **(C,F)** Single traces of MS2 intensity over nc14 from representative nuclei of indicated genotypes. **(G)** Fano factor (FF) of Pol II initiation events during nc13 (mean ± s.e.m.) calculated across a moving time window.

Statistics: sog^{WT} N= 5 embryos, n= 306 nuclei; $sog^{\Delta Dist}$ N= 5 embryos, n= 242 nuclei; $sog^{\Delta Prox}$ N= 4 embryos, n= 333 nuclei; sna^{WT} N= 5 embryos, n= 404 nuclei; $sna^{\Delta Dist}$ N= 4 embryos, n= 248 nuclei; $sna^{\Delta Prox}$ N= 5 embryos, n= 405 nuclei. Significance is indicated using Kruskal–Wallis test; ns = not significant. See Supplementary Movies 1-6.

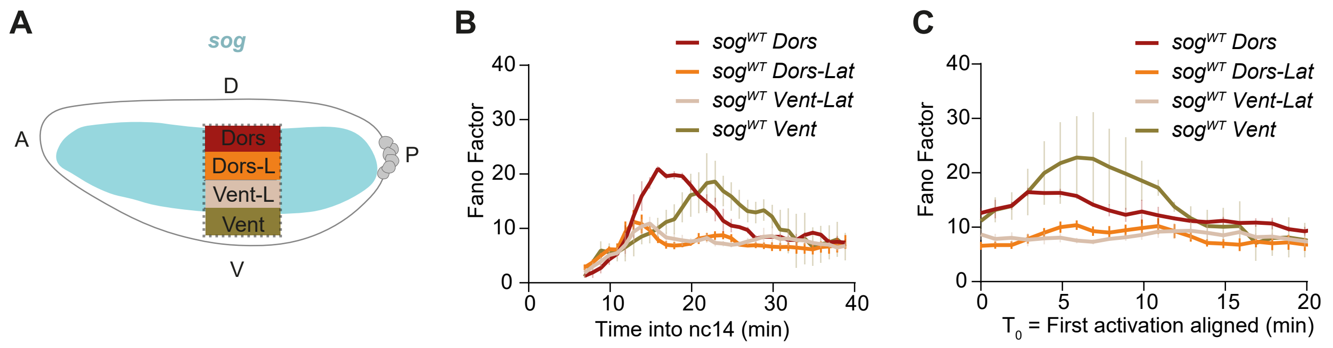

Fig. S2. Transcriptional dynamics of *sog-MS2 alleles* across different spatial domains (A) Schematic showing the domains of interest. **(B-C)** Fano factor (FF) of Pol II initiation events during nc14 (mean ± s.e.m. over embryos) within time windows: **(B)** after mitosis (T_0 = mitosis) or **(C)** after aligning the first activation of each nucleus to time T0.

Statistics: Dorsal region: *sog*^{WT} N= 2 embryos, n= 126 nuclei; *sog*$^{\Delta Prox}$ N= 4 embryos, n= 165 nuclei / Dorso-Lateral region: *sog*^{WT} N= 4 embryos, n= 286 nuclei; *sog*$^{\Delta Dist}$ N= 3 embryos, n= 84 nuclei; *sog*$^{\Delta Prox}$ N= 5 embryos, n= 407 nuclei / Ventral region: *sog*^{WT} N= 3 embryos, n= 140 nuclei; *sog*$^{\Delta Dist}$ N= 5 embryos, n= 220 nuclei; *sog*$^{\Delta Prox}$ N= 3 embryos, n= 121 nuclei; / Ventro-Lateral: *sog*^{WT} region N= 5 embryos, n= 306 nuclei. See Supplementary Movies 1-3.

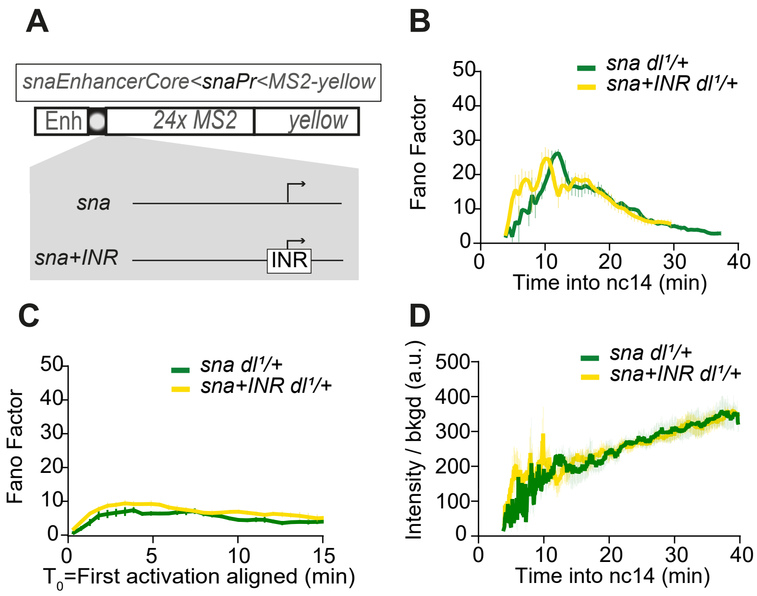

Fig. S3. Impact of INR promoter motif on transcriptional noise in a sensitized *dl* heterozygous genetic background (A) Schematic of the promoter transgenes used to decipher the impact of the INR motif (see also Supplementary Table 2). **(B-C)** Fano factor (FF) of Pol II initiation events during nc14 (mean ± s.e.m. over embryos) calculated across the moving time window: **(B)** after mitosis (T0 = mitosis) or **(C)** after aligning the first activation of each nucleus to time T0. Statistics: *sna* dl^1/+ N= 3 embryos, n= 181 nuclei ; *sna+INR* dl^1/+ N= 4 embryos, n= 220 nuclei.

Development • Supplementary information

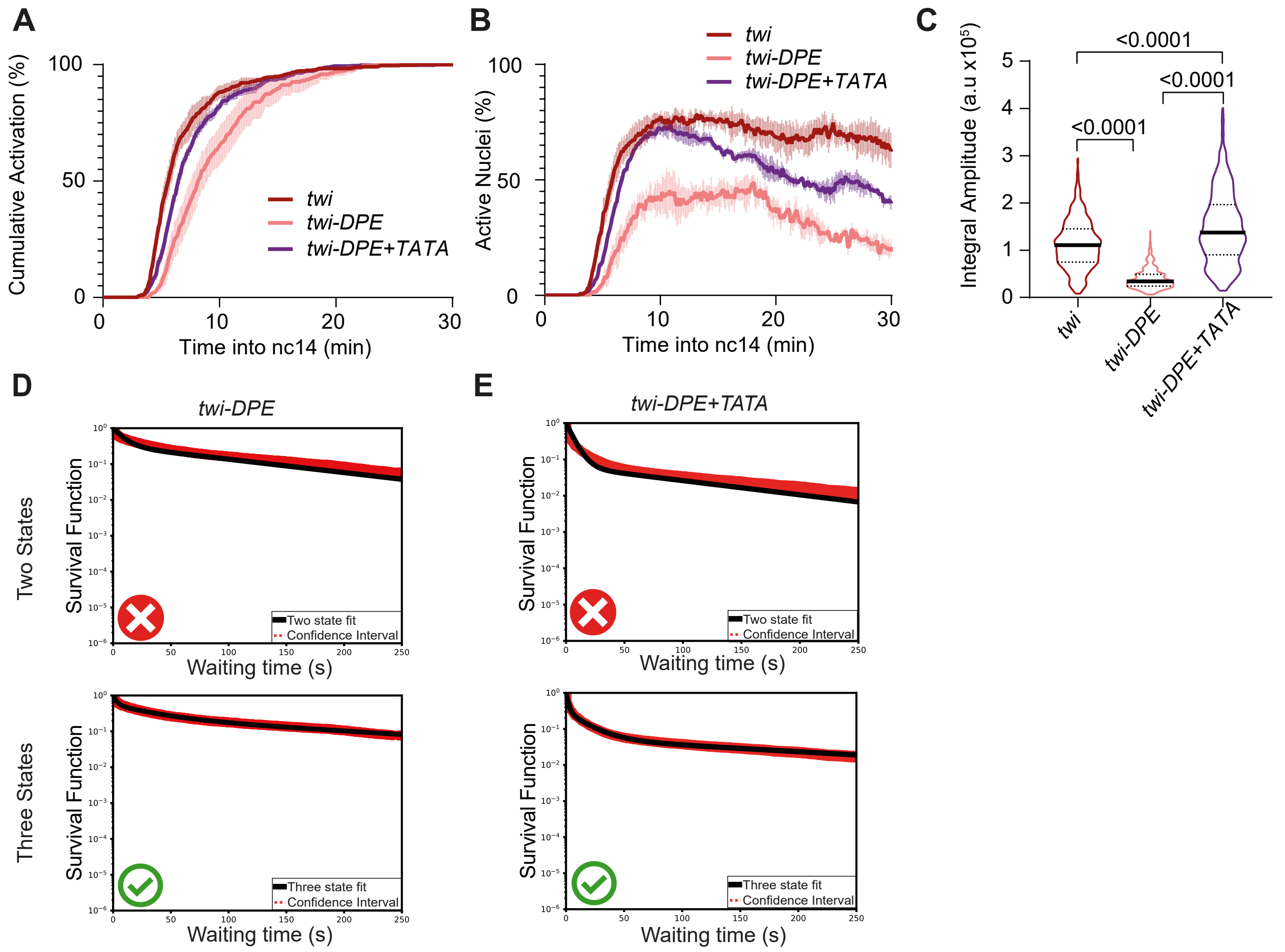

Fig. S4. Transcription dynamics from TATA and DPE containing promoters driven by a *twist* enhancer

(A) Cumulative activation percentage (mean ± s.e.m. over embryos) for the indicated domains and genotypes. **(B)** Percentage of active nuclei (mean ± s.e.m. over embryos) in nc14. **(C)** Distribution of AUC from intensity traces for the first 30 minutes of nc14. Solid line represents median, dashed lines are 1st and 3rd quartiles. **(D-E)** Survival function of the distribution of waiting times between polymerase initiation events (red circles) for the 20 to 30 min phase with the two-exponential (upper) and three-exponential (lower) population fittings estimated using the Kaplan–Meyer method (black line). The dashed lines indicate 95% confidence interval estimated based on Greenwood’s formula.

Statistics: *twi* N= 3 embryos, n= 485 nuclei; *twi-DPE* N= 3 embryos, n= 357 nuclei; *twi-DPE+TATA* N= 3 embryos, n= 451 nuclei. Significance is indicated using the Kruskal–Wallis test. See Supplementary Movies 7-11 and Table S3.

Development • Supplementary information

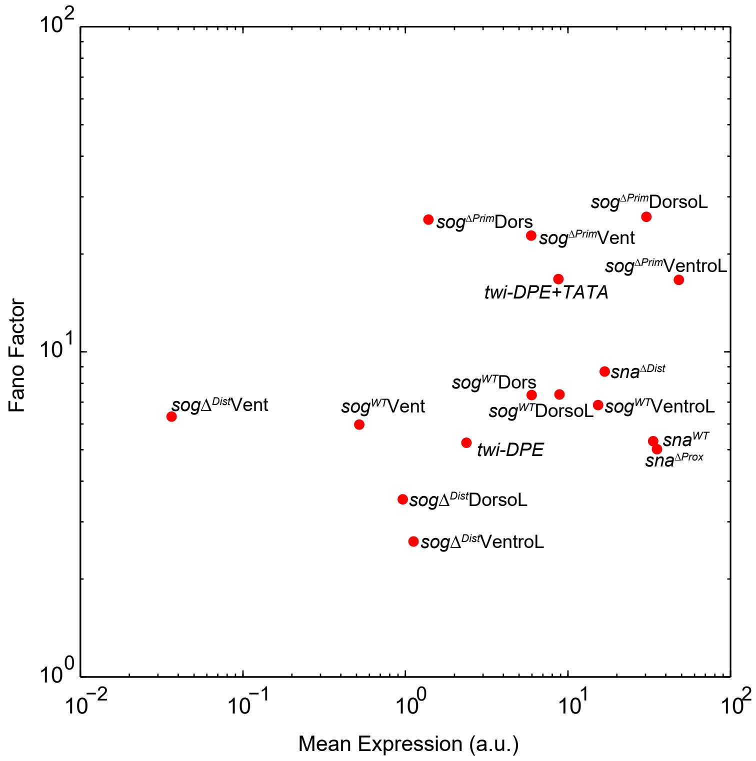

Fig. S5. Comparison of mean expression and Fano Factor for all tested genotypes and regions. VentroL: Ventro-Lateral; DorsoL: Dorso-Lateral; Dors: Dorsal; Vent:Ventral.

Development • Supplementary information

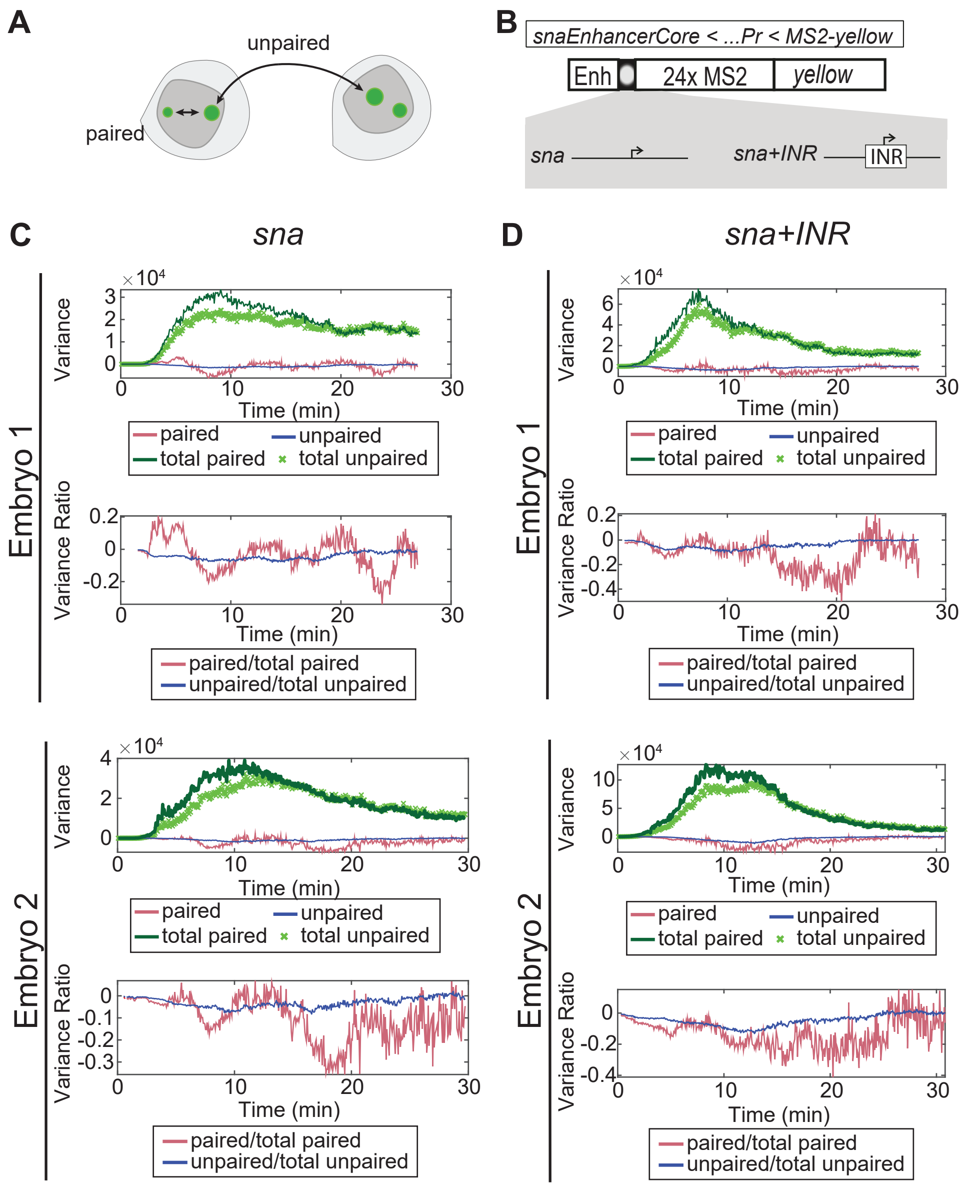

Fig. S6. A two-reporter construct is used to separate intrinsic from extrinsic noise. **(A)** Sites are considered *paired* when they are in the same nucleus and *unpaired* when they are randomly drawn from different nuclei. **(B)** Schema of the reporter constructs. **(C-D)** Decomposition of the noise variance for (C) *sna* and (D) *sna+INR*. The total variance is the sum of intrinsic and extrinsic noise and should be similar whether it is computed using paired or unpaired sites. The paired variance corresponds to extrinsic noise and is the sum of two components: intracellular and extracellular. The unpaired variance reflects only the extracellular component of extrinsic noise. Exact definitions of these components are provided in the Methods section.

Statistics: *sna* N= 2 embryos, n= 123 nuclei; *sna+INR* N= 2 embryos, n= 95 nuclei.

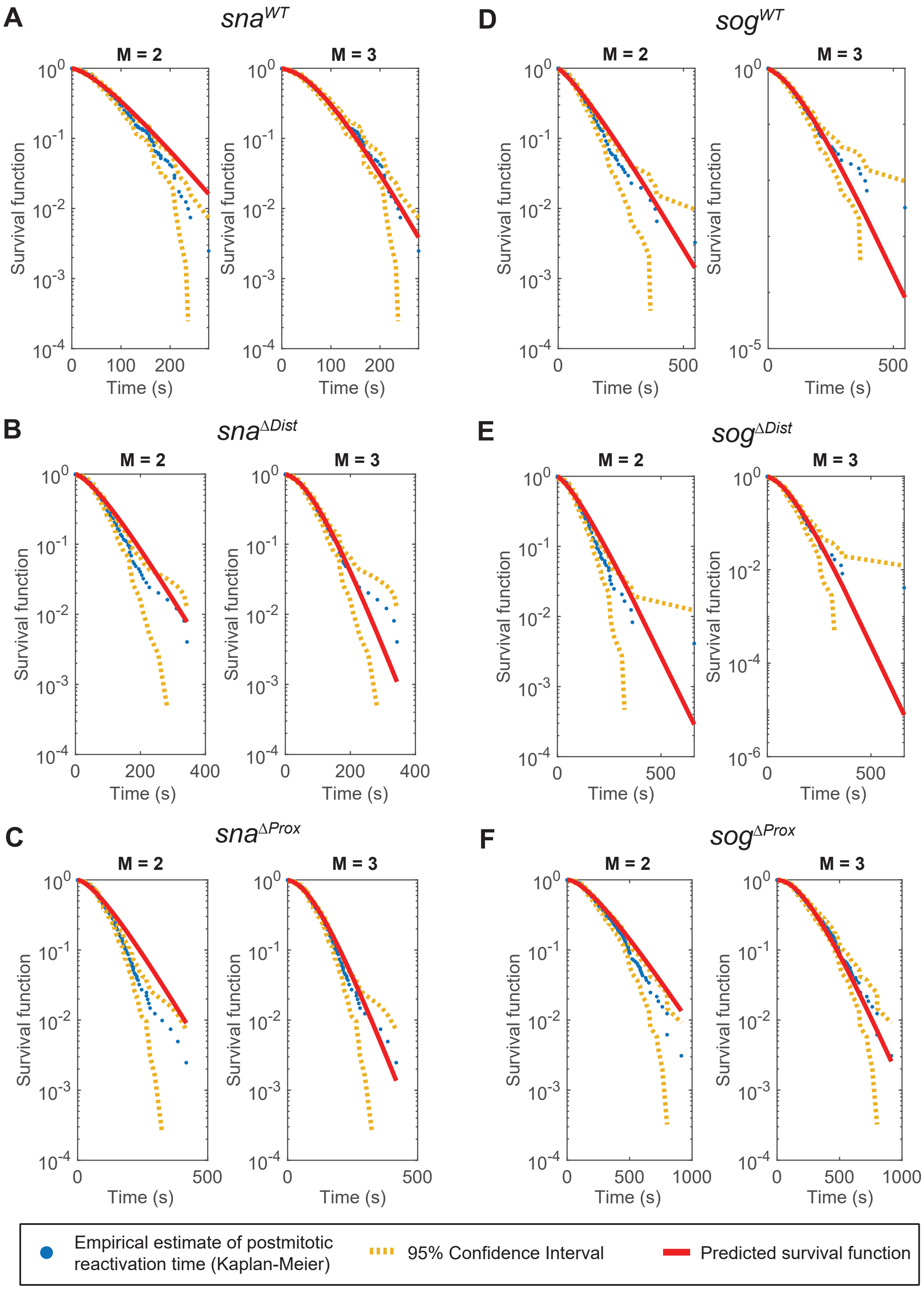

Fig. S7. A mixed Erlang model fitting of the distribution of postmitotic reactivation times. **(A-C)** Post-mitotic reactivation time fitting for sna^{WT} **(A)**, $sna^{\Delta Dist}$ **(B)** and $sna^{\Delta Prox}$ **(C)** for M=2 and M=3 steps. **(D-F)** Post-mitotic reactivation time fitting for sog^{WT} **(D)**, $sog^{\Delta Dist}$ **(E)** and $sog^{\Delta Prox}$ **(F)** for M=2 and M=3 steps. Empirical estimates of the survival function of these intervals using the Kaplan-Meier method are shown (blue) with 95% confidence intervals (orange) computed with Greenwood's formula and predicted survival function (red). A good fit occurs when the predicted survival function is within the bounds of the confidence interval. A good fit can be obtained by increasing the number of parameters in the mixed model, i.e. the maximal number of steps M.

Development • Supplementary information

Table S1. Fly lines associated with Maillard et al. (2025)

Line	Reference
; ; nos>MCP-eGFP, His2A-mRFP	Gift from T.Fukaya
sog ; ;	Whitney et al., Development 2022
sog$^{\Delta Proximal}$ *; ;*	Whitney et al., Development 2022
sog$^{\Delta Distal}$ *; ;*	Whitney et al., Development 2022
; sna^{WT} *;*	Bothma et al. eLife 2015
; sna$^{\Delta Proximal}$ *;*	Bothma et al. eLife 2015
; sna$^{\Delta Distal}$ *(shadow) ;*	Bothma et al. eLife 2015
; ; twi	This paper
; ; twi+DPE	This paper
; ; twi-DPE+TATA	This paper
*; **dl1** cn1 sca1/CyO, l(2)DTS1001;*	Bloomington 3236
;; sna	Pimmett et al Nat Commun 2021
;; sna+INR	Pimmett et al Nat Commun 2021
;; snaTATAlight	Pimmett et al Nat Commun 2021
;; Kr	Pimmett et al Nat Commun 2021
;; Kr-INR1	Pimmett et al Nat Commun 2021
;; Kr-INR2	Pimmett et al Nat Commun 2021
;; Kr-TATA	Pimmett et al Nat Commun 2021

Table S2. Promoter sequences for transgenes associated with Maillard et al. (2024)

Promoter name	Sequence
Twi	GAGCAGCCGCAAAATGTCAATTTGAGCAATGGCCGGAAGGATCCTGCGTCAGTTGCGTTCCGTAAGTGCGTGCGAGCAGATCGATCCAGCAAAACGCGGG
Twi ***-DPE***	GAGCAGCCGCAAAATGTCAATTTGAGCAATGGCCGGAAGGATCCTGCGTCAGTTGCGTTCCGTAAGTGCGTGCGAG**CCTCATGT**TCCAGCAAAACGCGGG
Twi ***-DPE +TATA***	GAGCAGCCGCAAAATGTCA**TATAAAAA**AATGGCCGGAAGGATCCTGCGTCAGTTGCGTTCCGTAAGTGCGTGCGAG**CCTCATGT**TCCAGCAAAACGCGGG
sna	GACAGCGGCGTCGGCAGAGGCGCAGAGTTCCGGGTATAAAAGAGCGTGCTCGACTGTTGACCTGTCACAGCCACCTCAGCTCTCGTTGAGAACGCAACCA
sna ***TATAlight***	GACAGCGGCGTCGGCAGAGGCGCAGAGTTCCGGG**TATAGTTG**AGCGTGCTCGACTGTTGACCTGTCACAGCCACCTCAGCTCTCGTTGAGAACGCAACCA
sna ***+INR***	GACAGCGGCGTCGGCAGAGGCGCAGAGTTCCGGGTATAAAAGAGCGTGCTCGACTGTTGACCTG**TCAGTC**CCACCTCAGCTCTCGTTGAGAACGCAACCA
kr	TTCGCCGAGACAGAGCGTACTTATAGTTAGCTCACGCAGCGAATTTGTATCAGTCGTGATTTGGCTCTGTCAGCGAAAGGAACAACCATTTGTTGTGCC
kr ***-INR1***	TTCGCCGAGACAGAGCGTACTTATAGTTAGCTCACGCAGCGAATTTGTA**TCACAG**GTGATTTGGCTCTGTCAGCGAAAGGAACAACCATTTGTTGTGCC
kr ***-INR2***	TTCGCCGAGACAGAGCGTACTTATAGTTAGCTCACGCAGCGAATTTGTA**CAATTG**GTGATTTGGCTCTGTCAGCGAAAGGAACAACCATTTGTTGTGCC
kr ***-TATA***	TTCGCCGAGACAGAGCGTAC**TGCACAAA**GGCTCACGCAGCGAATTTGTATCAGTCGTGATTTGGCTCTGTCAGCGAAAGGAACAACCATTTGTTGTGCC

Development • Supplementary information

Table S3. *twi* transgene kinetic parameters associated with Maillard et al. (2025)

twi-DPE														
	k_1^p	k_1^m	k_2^p	k_2^m	k_{ini}	p(OFF1)	p(OFF2)	p(ON)	T(OFF1) (s)	T(OFF2) (s)	T(ON) (s)	T(ini) (s)	Objective Function	Kolmogorov-Smirnov test
Optimal fitting	0.006	0.041	0.048	0.114	0.155	0.678	0.227	0.095	175.249	20.857	6.464	6.451		
CI Minimum	0.006	0.041	0.048	0.114	0.155	0.678	0.227	0.095	175.245	20.855	6.463	6.450		
CI Maximum	0.006	0.041	0.048	0.114	0.155	0.678	0.227	0.095	175.250	20.858	6.464	6.451	0.002	0.996

twi-DPE+TATA														
	k_1^p	k_1^m	k_2^p	k_2^m	k_{ini}	p(OFF1)	p(OFF2)	p(ON)	T(OFF1) (s)	T(OFF2) (s)	T(ON) (s)	T(ini) (s)	Objective Function	Kolmogorov-Smirnov test
Optimal fitting	0.004	0.020	0.077	0.106	0.373	0.658	0.198	0.144	226.847	12.978	7.950	2.681		
CI Minimum	0.004	0.020	0.077	0.106	0.373	0.658	0.198	0.144	226.846	12.978	7.949	2.681		
CI Maximum	0.004	0.020	0.077	0.106	0.373	0.658	0.198	0.144	226.848	12.978	7.950	2.681	0.003	1.000

Development • Supplementary information

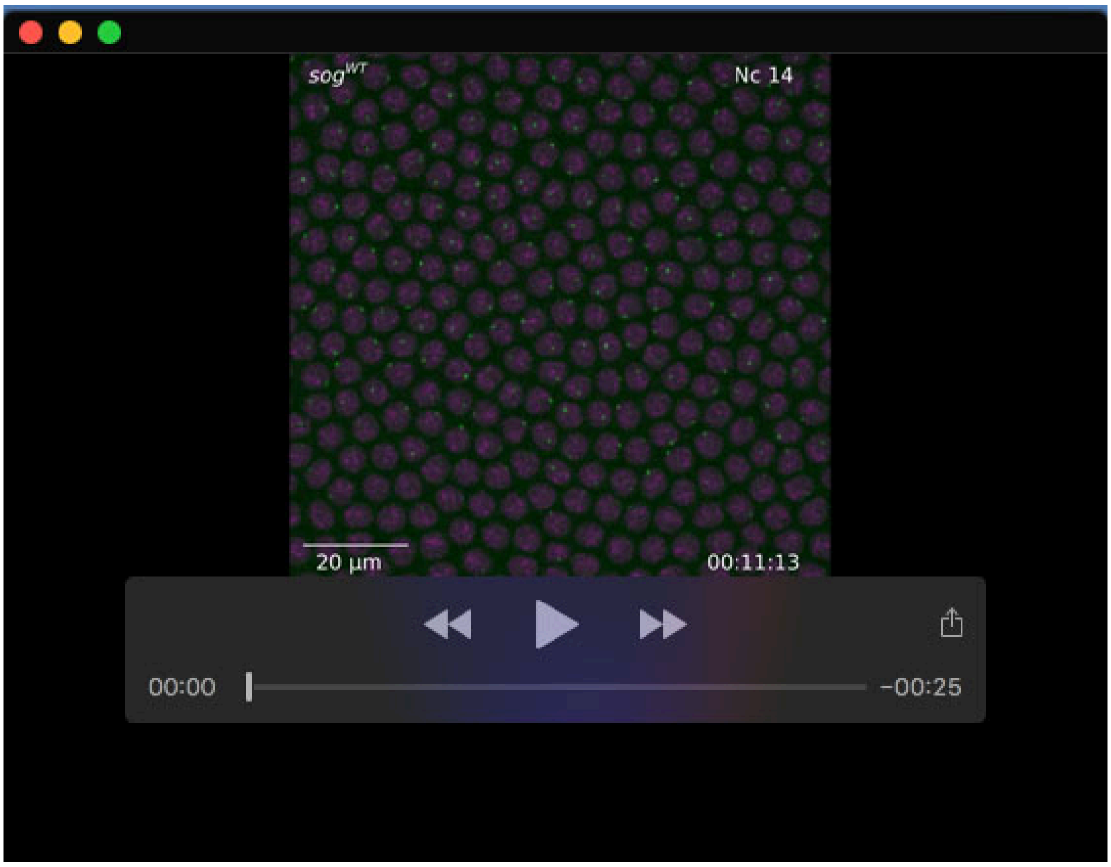

Movie 1. Live imaging of *sog*^{WT} representative of nc13-14 beginning at mitosis (lateral view). MS2-tag are detected using MCP-eGFP (green) and nuclei using His2Av-mRFP (magenta) using a Zeiss 880 fast Airyscan confocal microscope, with Z-stacks acquired at 6.35-second intervals.

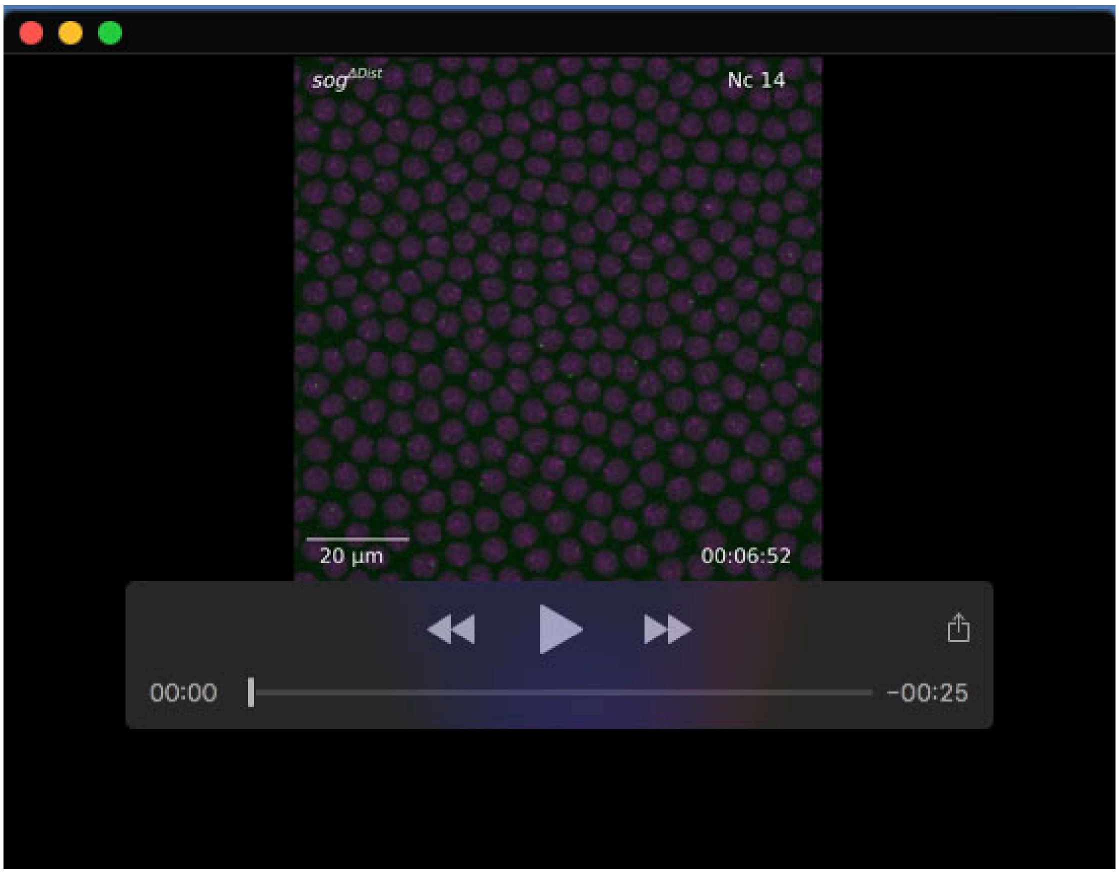

Movie 2. Live imaging of *sog*$^{\Delta Dist}$ representative of nc13-14 beginning at mitosis (lateral view). MS2-tag are detected using MCP-eGFP (green) and nuclei using His2Av-mRFP (magenta) using a Zeiss 880 fast Airyscan confocal microscope, with Z-stacks acquired at 6.35-second intervals.

Development • Supplementary information

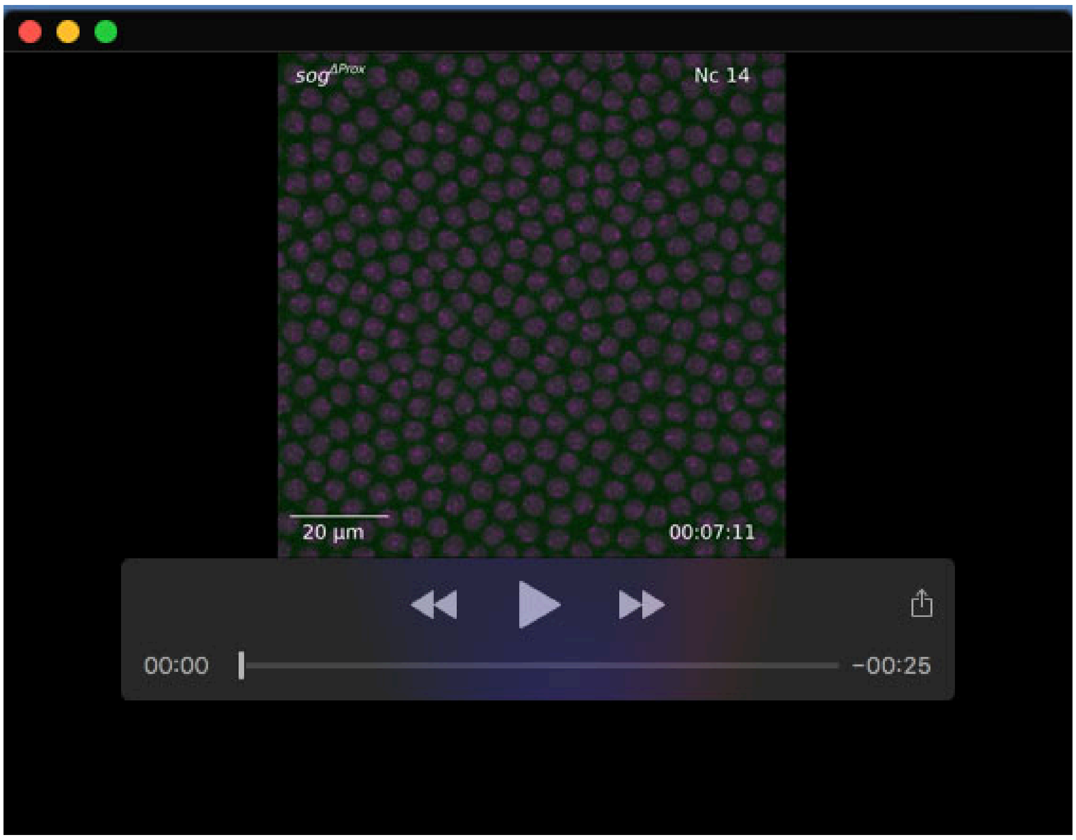

Movie 3. Live imaging of *sog*$^{\Delta Prox}$ representative of nc13-14 beginning at mitosis (lateral view). MS2-tag are detected using MCP-eGFP (green) and nuclei using His2Av-mRFP (magenta) using a Zeiss 880 fast Airyscan confocal microscope, with Z-stacks acquired at 6.35-second intervals.

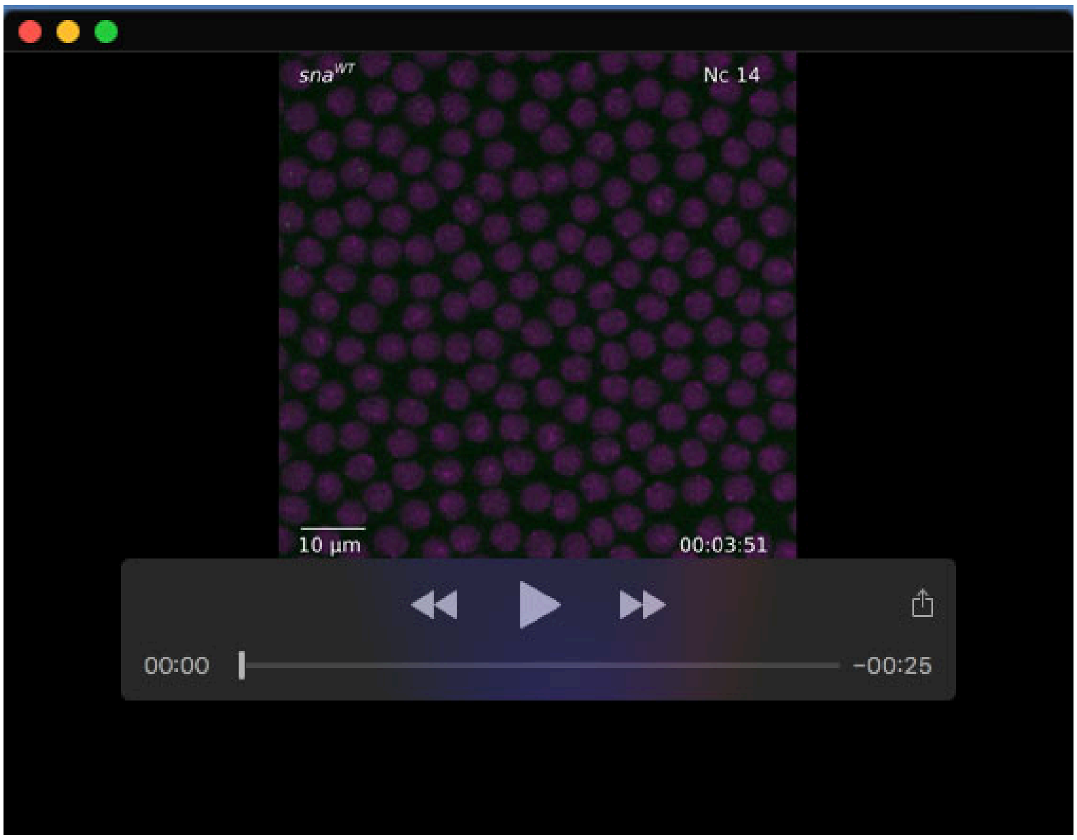

Movie 4. Live imaging of *sna*^{WT} representative of nc13-14 beginning at mitosis (lateral view). MS2 are detected using MCP-eGFP (green) and nuclei using His2Av-mRFP (magenta) using a Zeiss 880 fast Airyscan confocal microscope, with Z-stacks acquired at 4.64-second intervals.

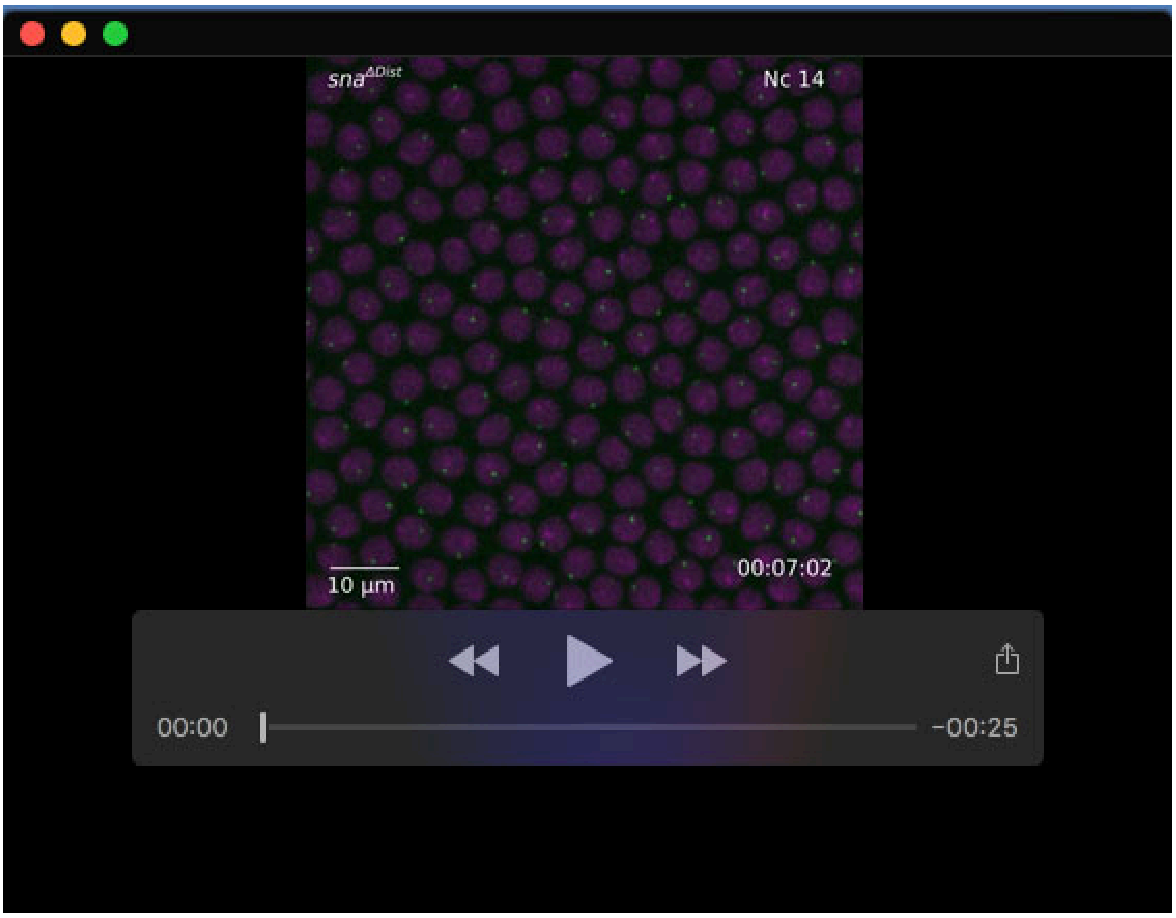

Movie 5. Live imaging of *sna*$^{\Delta Dist}$ representative of nc13-14 beginning at mitosis (ventral view). MS2 are detected using MCP-eGFP (green) and nuclei using His2Av-mRFP (magenta) using a Zeiss 880 fast Airyscan confocal microscope, with Z-stacks acquired at 4.64-second intervals.

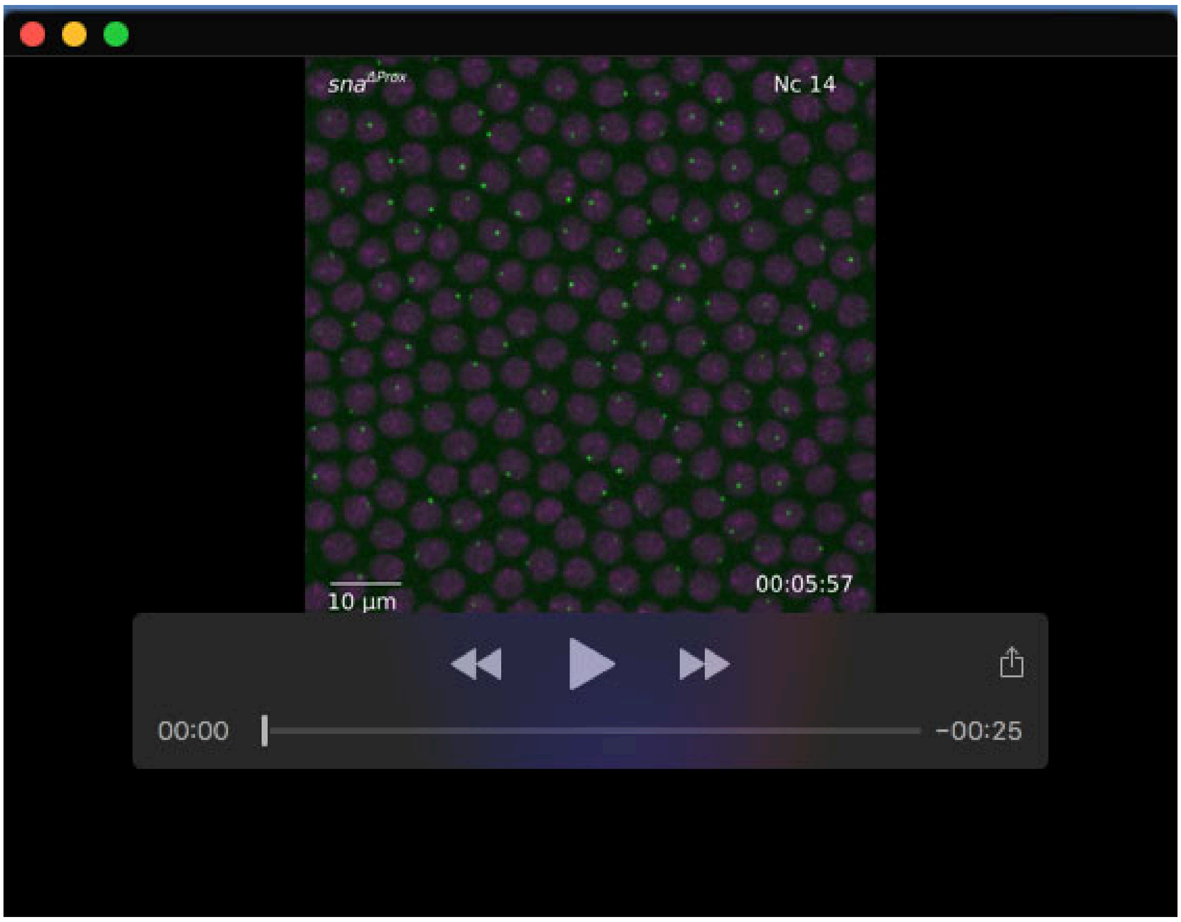

Movie 6. Live imaging of *sna*$^{\Delta Prox}$ representative of nc13-14 beginning at mitosis (ventral view). MS2 are detected using MCP-eGFP (green) and nuclei using His2Av-mRFP (magenta) using a Zeiss 880 fast Airyscan confocal microscope, with Z-stacks acquired at 4.64-second intervals.

Development • Supplementary information

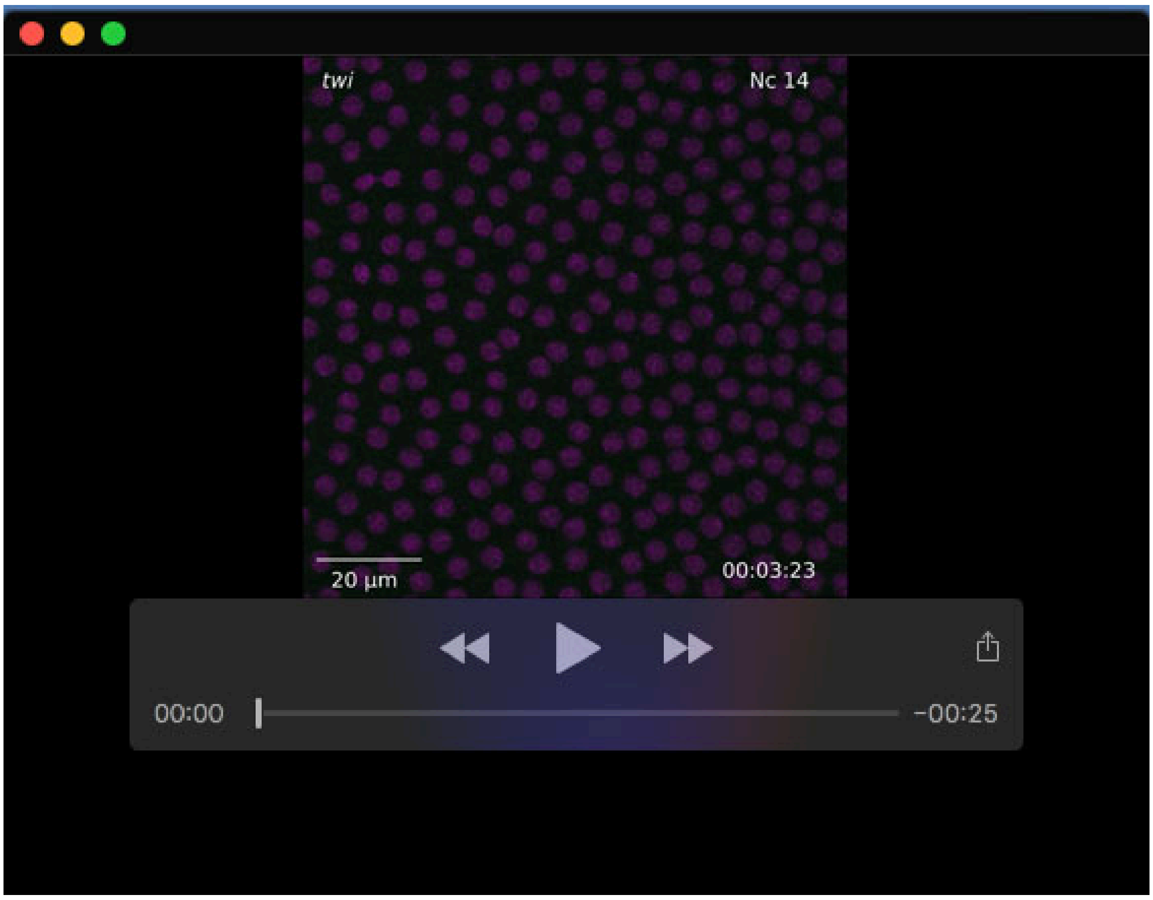

Movie 7. Live imaging of *twi* representative of nc13-14 beginning at mitosis (ventral view). MS2 are detected using MCP-eGFP (green) and nuclei using His2Av-mRFP (magenta) using a Zeiss 880 fast Airyscan confocal microscope, with Z-stacks acquired at 6.35-second intervals.

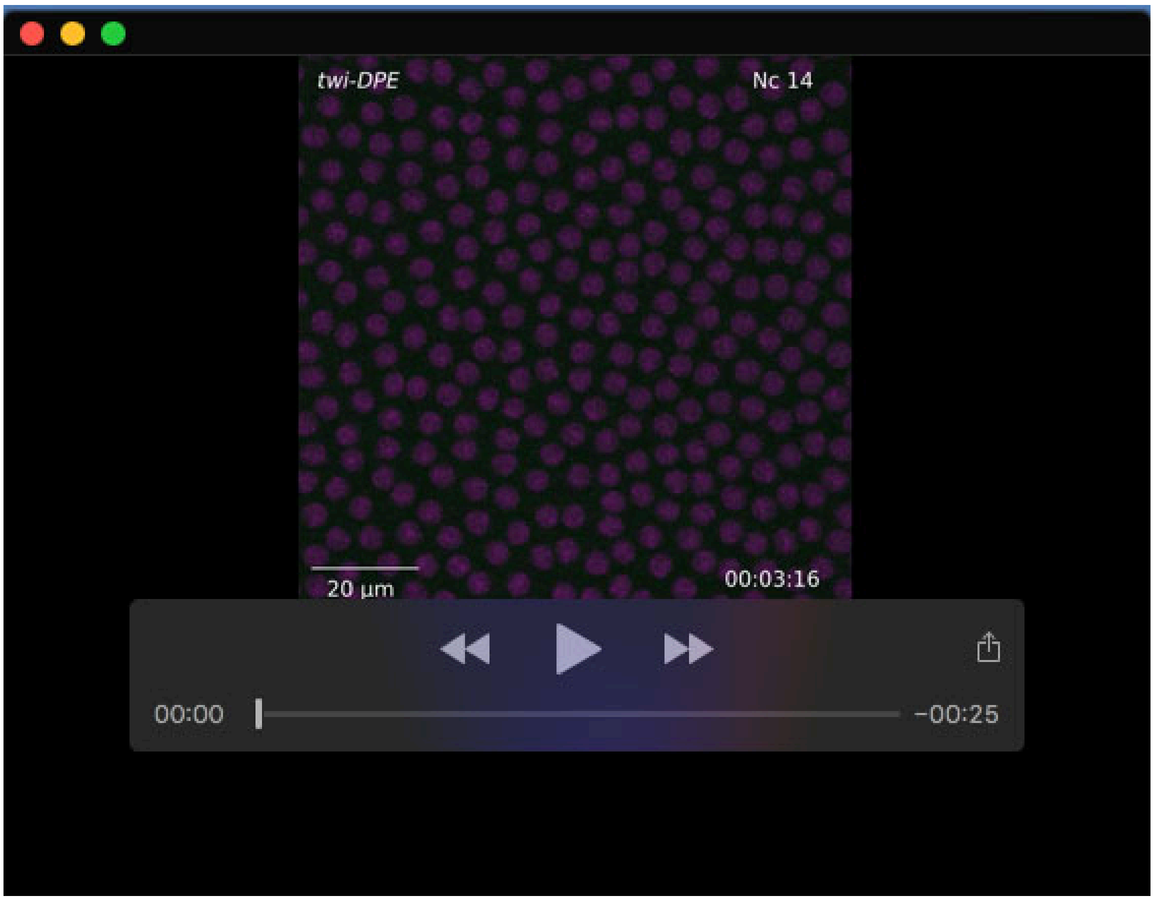

Movie 8. Live imaging of *twi-DPE* representative of nc13-14 beginning at mitosis (ventral view). MS2 are detected using MCP-eGFP (green) and nuclei using His2Av-mRFP (magenta) using a Zeiss 880 fast Airyscan confocal microscope, with Z-stacks acquired at 6.35-second intervals.

Development • Supplementary information

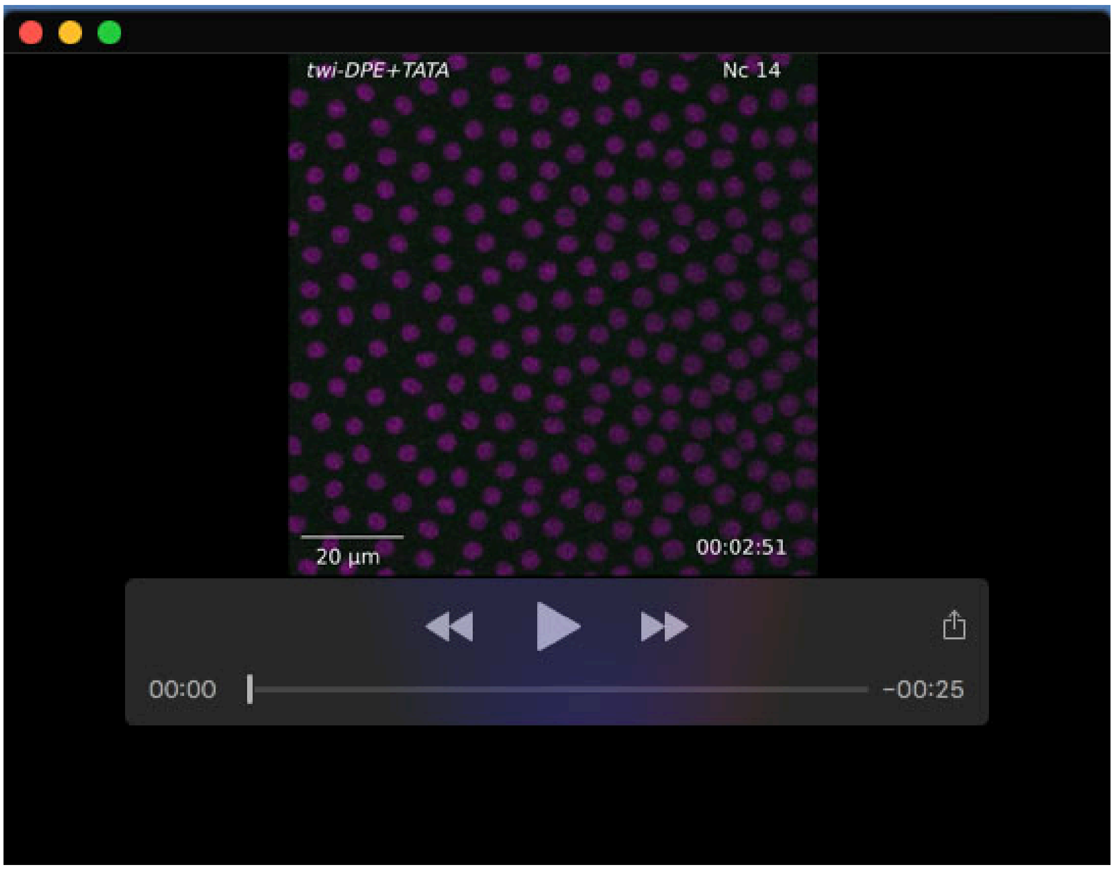

Movie 9. Live imaging of *twi-DPE+TATA* representative of nc13-14 beginning at mitosis (ventral view). MS2 are detected using MCP-eGFP (green) and nuclei using His2Av-mRFP (magenta) using a Zeiss 880 fast Airyscan confocal microscope, with Z-stacks acquired at 6.35-second intervals.

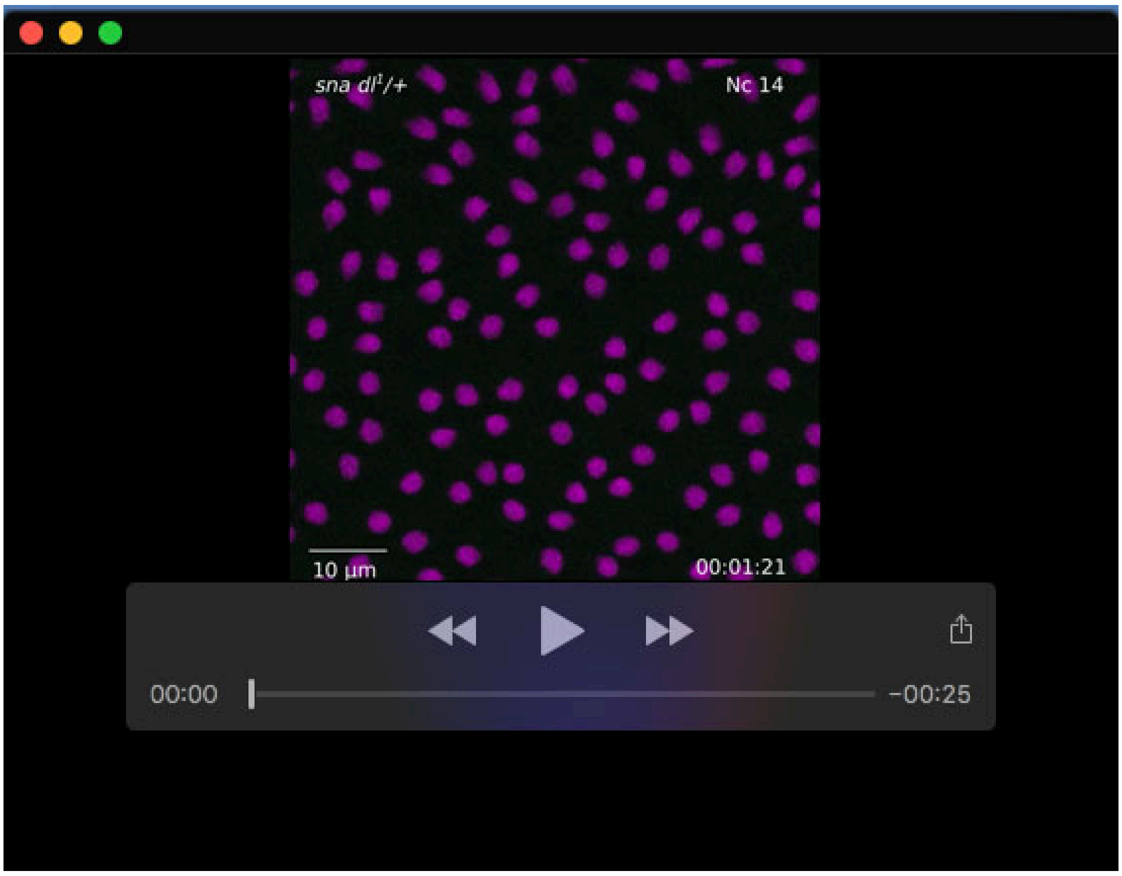

Movie 10. Live imaging of *sna* in *dl*^{1}/+ background representative of 14 beginning at mitosis (ventral view). MS2 are detected using MCP-eGFP (green) and nuclei using His2Av-mRFP (magenta)using a Zeiss 880 fast Airyscan confocal microscope, with Z-stacks acquired at 3.86-second intervals.

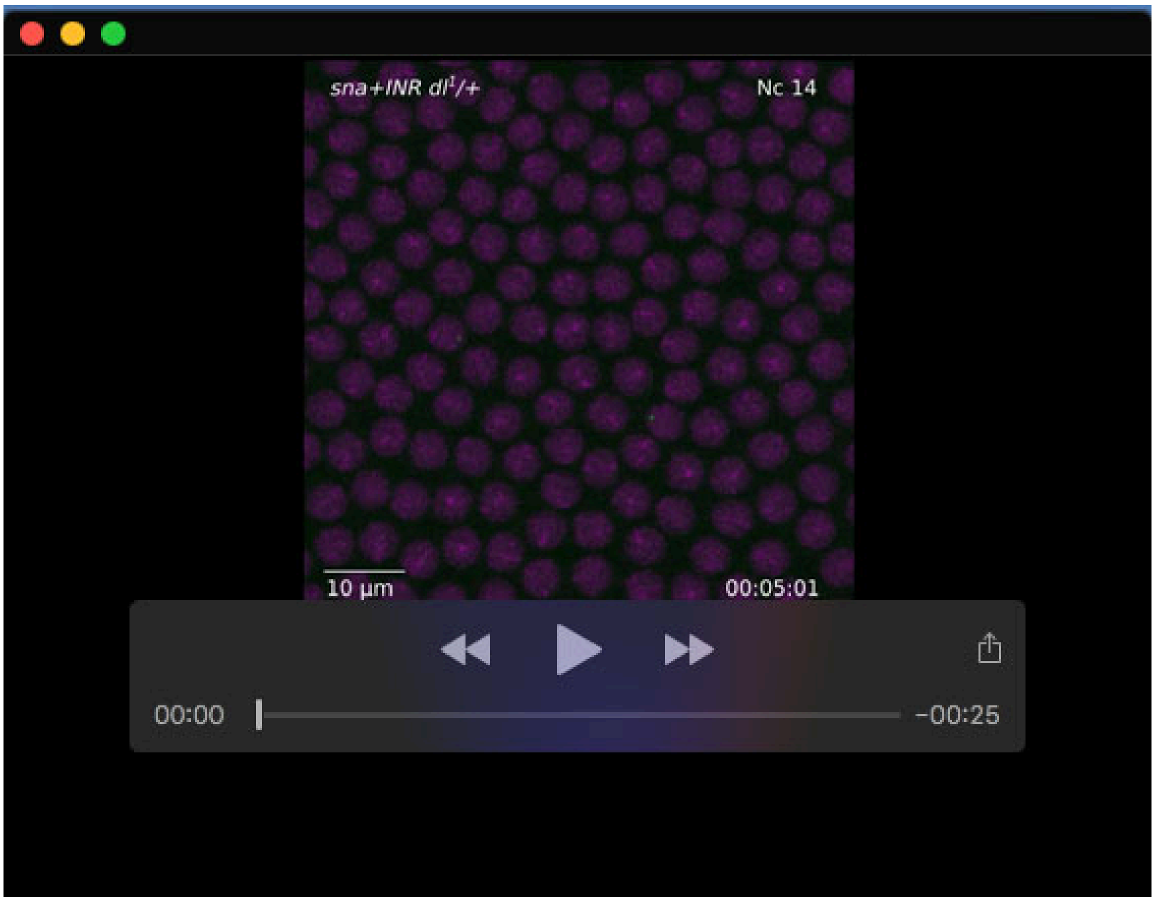

Movie 11. Live imaging of *sna+INR* in *dl^1/+* background representative of nc14 beginning at mitosis (ventral view). MS2 are detected using MCP-eGFP (green) and nuclei using His2Av-mRFP (magenta) using a Zeiss 880 fast Airyscan confocal microscope, with Z-stacks acquired at 3.86-second intervals.

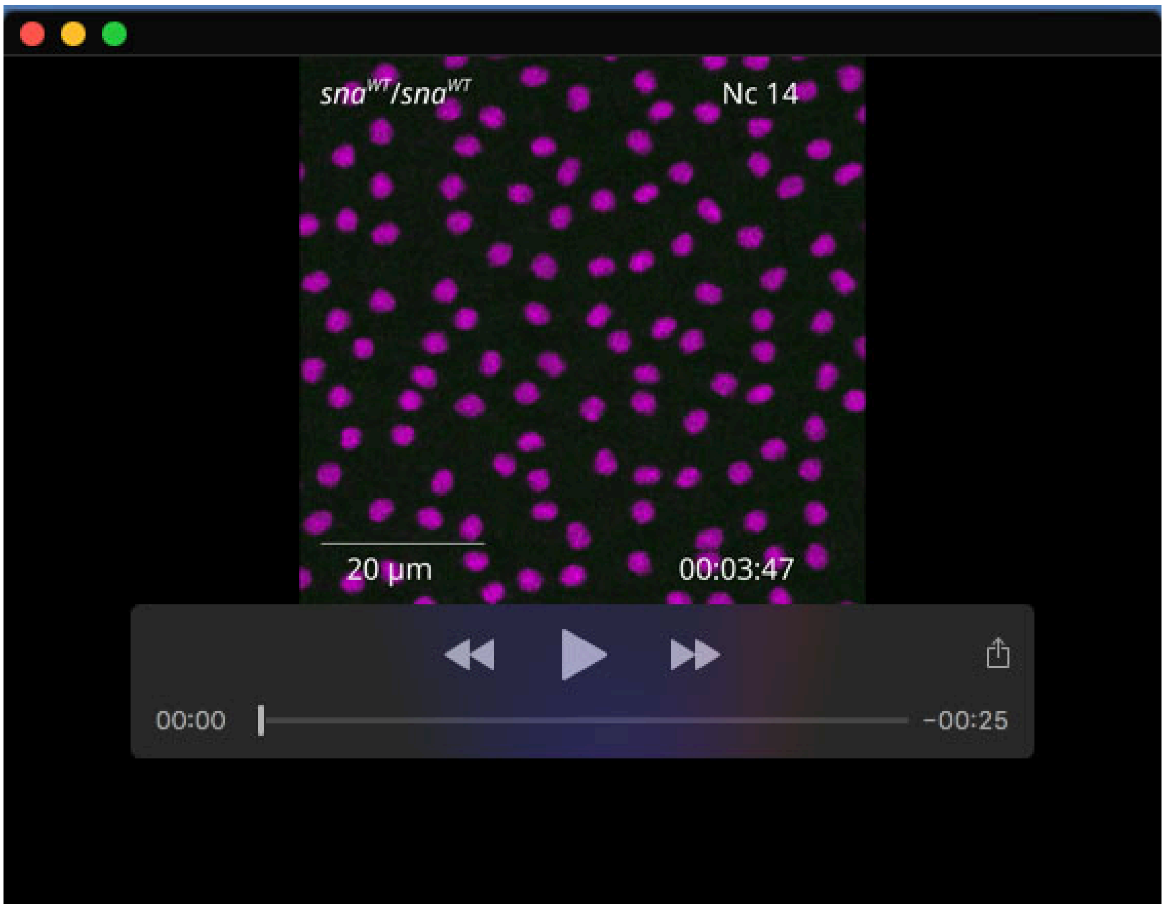

Movie 12. Live imaging of *sna/sna* representative of nc14 beginning at mitosis (ventral view). MS2 are detected using MCP-eGFP (green) and nuclei using His2Av-mRFP (magenta) using a Zeiss 880 fast Airyscan confocal microscope, with Z-stacks acquired at 3.86-second intervals.

Supplementary Materials and Methods

Computing the Fano Factor of the Number of Transcription Initiation Events in a Fixed Time Window at Steady State for Two-State and Three-State Models

1 Using renewal theory to relate the Fano factor to moments of the distribution of waiting times between successive initiation events at the steady state

The times of successive initiation events generated by a bursting promoter can be modeled as a renewal process [2]. Renewal processes generalize the Poisson process by allowing the waiting time between successive events to be non–exponentially distributed. However, as for Poisson processes, successive waiting times are assumed to be independent. At steady state, which we assume is satisfied here, the distribution of waiting times is constant.

Under these assumptions, it can be shown [1] that, for sufficiently large observation windows of length T, both the variance and the mean of the number of events N scale linearly with T:

$$\mathrm{Var}(N) = T\,\sigma^2, \qquad \langle N\rangle = T\,\mu, \tag{S1}$$

where σ and μ denote the standard deviation and the mean of the waiting time between successive events. For a Poisson process, the waiting time is exponentially distributed, so that $\sigma = \mu$, but in general σ and μ are independent.

From Eq. (S1), we obtain

$$\mathrm{Fano}(N) = \frac{\mathrm{Var}(N)}{\langle N\rangle} = \left(\frac{\sigma}{\mu}\right)^2, \tag{S2}$$

which shows that the Fano factor does not depend on the window length T, provided T is sufficiently large.

Development • Supplementary information

2 Relating the Fano factor to kinetics parameters

For a bursting model with N states, the waiting-time distribution between successive initiation events is multiexponential and is characterized by the survival function

$$S(t) = \mathbb{P}(\tau > t) = A_1 e^{\lambda_1 t} + \cdots + A_N e^{\lambda_N t}, \tag{S3}$$

where $\sum_{i=1}^{N} A_i = 1$ and $\lambda_i < 0$ for $i = 1, \ldots, N$ [3, 2].

One then has the following relations [3]:

$$\mu = -\left(\frac{A_1}{\lambda_1} + \cdots + \frac{A_N}{\lambda_N}\right), \tag{S4}$$

$$\sigma^2 = 2\left(\frac{A_1}{\lambda_1^2} + \cdots + \frac{A_N}{\lambda_N^2}\right) - \mu^2. \tag{S5}$$

The parameters A_i and λ_i can be related to the bursting parameters. For several models with two, three, four, and more states we computed the bursting parameters from A_i and λ_i using computer algebra methods [2, 3]. However, computing A_i and λ_i from kinetic parameters in order to express σ, μ, and Fano as functions of the kinetics parameters, is not possible in general.

Doing this for five states or more demands solving polynomial equations of degree five or more symbolically, which is not possible by the Abel-Rufini theorem. Even for three and four states, the resulting analytical formulas can be complex and impractical to use. We show that it is possible to obtain simplified analytical formulas for σ, μ and Fano, without having to compute both A_i and λ_i.

We present this procedure for two models: the two-state (telegraph) model and the three-state model illustrated in Fig. 6D (Model M9 in [2]). It is possible that the procedure can be generalized but this is beyond the scope of these notes.

In a first step we express the coefficients A_i as functions of the kinetic parameters and exponents λ_i. This can be obtained from a system of linear equations for symmetric combinations of A_i and powers of λ_i.

For the three state model, we use the following three equations to find A_1,A_2,A_3:

$$-\left(\frac{A_1}{\lambda_1} + \frac{A_2}{\lambda_2} + \frac{A_3}{\lambda_3}\right) = \frac{k_1^{\text{on}} k_2^{\text{on}} + k_1^{\text{on}} k_2^{\text{off}} + k_2^{\text{on}} k_1^{\text{off}}}{k_1^{\text{on}} k_2^{\text{on}} k_{\text{ini}}} \tag{S6}$$

$$-\left(A_1\lambda_1 + A_2\lambda_2 + A_3\lambda_3\right) = k_{\text{ini}} \tag{S7}$$

$$A_1 + A_2 + A_3 = 1, \tag{S8}$$

Development • Supplementary information

The r.h.s. of (S6) is simply $1/(P_{ON}k_{ini})$, as $\mu = 1/(P_{ON}k_{ini})$ (see Table 1 in [2]),

$$P_{\mathrm{ON}}k_{\mathrm{ini}} = \frac{k_1^{\mathrm{on}}k_2^{\mathrm{on}}k_{\mathrm{ini}}}{k_1^{\mathrm{on}}k_2^{\mathrm{on}} + k_1^{\mathrm{on}}k_2^{\mathrm{off}} + k_2^{\mathrm{on}}k_1^{\mathrm{off}}}. \tag{S9}$$

(S7) is part of the symmetrized algebraic system relating A_i, λ_i and the kinetic coefficients (see Sections 3.4, 3.5 of [2]). Finally, (S8) is a general property of the coefficients A_i (coming from the fact that the survival function $S(t)$ satisfies $S(0) = 1$ by definition).

In a second step, we use the solutions of the linear system (S6),(S7),(S8) to compute the Fano factor (see also (S2),(S4),(S5))

$$\mathrm{Fano} = 2\frac{\frac{A_1}{\lambda_1^2} + \frac{A_2}{\lambda_2^2} + \frac{A_3}{\lambda_3^2}}{\left(\frac{A_1}{\lambda_1} + \frac{A_2}{\lambda_2} + \frac{A_3}{\lambda_3}\right)^2} - 1. \tag{S10}$$

It turns out that the resulting expression depends on $\lambda_1, \lambda_2, \lambda_3$ via elementary symmetric polynomials of degree at most three:

$$\begin{aligned}\mathrm{Fano} = 1 - \Big[2\Big(&(k_1^{on}(k_2^{on})^2k_{ini}^3 + (k_1^{on}(k_2^{on}k_{ini})^2L_1 + ((k_1^{on}(k_2^{on})^2 + k_1^{on}(k_2^{on})^2k_1^{off}k_{ini} + k_1^{on}(k_2^{on})^2k_1^{off}k_{ini})L_2+\\ &+((k_1^{on}k_2^{on})^2 + (k_1^{on}k_2^{off})^2 + (k_2^{on}k_1^{off})^2 + 2k_1^{on}(k_2^{on})^2k_1^{off} + 2(k_1^{on})^2k_2^{on}k_2^{off} + 2k_1^{on}k_2^{on}k_1^{off}k_2^{off})\Big)\Big]/\\ &/\Big[L_3\big(k_1^{on}k_2^{on} + k_1^{on}k_2^{off} + k_2^{on}k_1^{off}\big)^2\Big],\end{aligned} \tag{S11}$$

where $L_1 = \lambda_1 + \lambda_2 + \lambda_3$, $L_2 = \lambda_1\lambda_2 + \lambda_1\lambda_3 + \lambda_2\lambda_3$, $L_3 = \lambda_1\lambda_2\lambda_3$ are elementary symmetric polynomials.

L_1,L_2,L_3 can be related to kinetic parameters using the Vieta's formulas (see Eqs.(13),(19-21) of [2]):

$$L_1 = -k_1^{on} - k_2^{on} - k_1^{off} - k_2^{off} - k_{ini}, \tag{S12}$$

$$L_2 = k_1^{on}k_2^{on} + k_1^{on}k_2^{off} + k_1^{on}k_{ini} + k_2^{on}k_1^{off} + k_2^{on}k_{ini}, \tag{S13}$$

$$L_3 = -k_1^{on}k_2^{on}k_{ini}. \tag{S14}$$

It follows

$$\mathrm{FANO} = 1 + \frac{2k_{\mathrm{ini}}\left(k_2^{\mathrm{off}}(k_1^{\mathrm{on}})^2 + k_1^{\mathrm{off}}(k_2^{\mathrm{on}})^2\right)}{\left(k_1^{\mathrm{on}}k_2^{\mathrm{on}} + k_1^{\mathrm{on}}k_2^{\mathrm{off}} + k_2^{\mathrm{on}}k_1^{\mathrm{off}}\right)^2}. \tag{S15}$$

We use the same procedure to compute Fano for a two-state model.

First, we solve the system

$$-(A_1/\lambda_1 + A_2/\lambda_2) = \frac{k_{on} + k_{off}}{k_{on}k_{ini}} \tag{S16}$$

$$A_1 + A_2 = 1, \tag{S17}$$

where we have used

$$P_{\mathrm{ON}}k_{\mathrm{ini}} = \frac{k_{\mathrm{on}}k_{\mathrm{ini}}}{k_{\mathrm{on}} + k_{\mathrm{off}}}. \tag{S18}$$

Development • Supplementary information

Then, we compute the FANO factor

$$\text{Fano} = 2\frac{\frac{A_1}{\lambda_1^2} + \frac{A_2}{\lambda_2^2}}{\left(\frac{A_1}{\lambda_1} + \frac{A_2}{\lambda_2}\right)^2} - 1.$$

It follows

$$\text{Fano} = 1 - 2\frac{k_{ini}^2 k_{on}^2 + \left(k_{ini} k_{on}^2 + k_{ini} k_{off} k_{on}\right) L_1 + \left(k_{off} + k_{on}\right)^2 L_2}{L_2 (k_{off} + k_{on})^2}. \tag{S19}$$

Using the Vieta's formulas

$$L_1 = -\left(k_{ini} + k_{on} + k_{off}\right), \tag{S20}$$

$$L_2 = k_{on}\, k_{ini}, \tag{S21}$$

we obtain

$$\text{FANO} = 1 + \frac{2k_{\text{ini}} k_{\text{off}}}{(k_{\text{off}} + k_{\text{on}})^2}. \tag{S22}$$

These symbolic calculations, together with a numerical example, are available as a Matlab code at `https://github.com/oradules/fano4transcription`

References

[1] William Feller. *An Introduction to Probability Theory and its Applications, Volume 1.* John Wiley and sons, New York, 1968.

[2] Ovidiu Radulescu, Dima Grigoriev, Matthias Seiss, Maria Douaihy, Mounia Lagha, and Edouard Bertrand. Identifying markov chain models from time-to-event data: an algebraic approach. *Bulletin of Mathematical Biology*, 87(1):11, 2025.

[3] Katjana Tantale, Encar Garcia-Oliver, Marie-Cécile Robert, Adèle L'Hostis, Yueyuxiao Yang, Nikolay Tsanov, Rachel Topno, Thierry Gostan, Alja Kozulic-Pirher, Meenakshi Basu-Shrivastava, Kamalika Mukherjee, Vera Slaninova, Jean-Christophe Andrau, Florian Mueller, Eugenia Basyuk, Ovidiu Radulescu, and Edouard Bertrand. Stochastic pausing at latent HIV-1 promoters generates transcriptional bursting. *Nature Communications*, 12(1):4503, Jul 2021.
